# Uterine torsion with fetal death in the third trimester: Case report

**DOI:** 10.1097/MD.0000000000043810

**Published:** 2025-08-15

**Authors:** Linghui Liu, Min Zhou, Yanting Ding

**Affiliations:** a Department of Gynaecology and Obstetrics, Xiang Yang Central Hospital, Affliated Hospital of Hubei University of Arts and Science, Xiangyang, China.

**Keywords:** case report, fetal death, obstetric complication, uterine torsion

## Abstract

**Rationale:**

Uterine torsion is a rare obstetric complication that can easily lead to fetal death. The sudden onset and atypical clinical presentation make diagnosis very difficult.

**Patient concerns:**

We reported a case of uterine torsion in the third trimester of pregnancy, which was mainly characterized by severe abdominal pain and shock.

**Diagnoses:**

The diagnosis of this case was confirmed through surgery. Neither preoperative ultrasound nor computed tomography could provide a definite diagnosis.

**Interventions:**

Anti-shock therapy and emergency cesarean section were promptly initiated. The uterus was repositioned to its normal anatomical position, followed by the delivery of a deceased baby via a standard cesarean section. Additionally, bilateral round ligament of the uterus was surgically shortened.

**Outcomes:**

The abdominal pain and shock of the patient were completely relieved postoperatively, and she was discharged 4 days later. Uterus morphology and position recovered well on the 14th and 42nd day after surgery.

**Lessons:**

The rarity of uterine torsion during pregnancy and the limited clinical experience in its management underscore the clinical significance of documenting each case for improved understanding and evidence-based practice. Uterine torsion must be included in the differential diagnosis when acute abdominal pain and hemodynamic instability occur during pregnancy, particularly in cases where conventional etiologies (e.g., uterine rupture, placental abruption) fail to account for the clinical presentation. Presumptive diagnoses of uterine torsion necessitate expeditious surgical exploration, as time-critical intervention is paramount to mitigate catastrophic maternal–fetal complications including hemorrhagic shock and irreversible ischemic injuries.

## 1. Introduction

Uterine torsion is a rare obstetric complication, defined as the rotation of the uterus by more than 45° along the major axis.^[1–3]^ The clinical manifestations are atypical and varied, making a definitive diagnosis difficult.^[4]^ The majority of patients are detected during surgical explorations. In this case report, we present a case of uterine torsion resulting in stillbirth at 33 weeks and 3 days of gestational age.

## 2. Case report

A 28-year-old pregnant woman, gravida 2, para 1, at 33 weeks and 3 days gestational age, was admitted to the hospital on August 15, 2024, due to acute paroxysmal abdominal pain accompanied by diaphoresis. Her obstetric history included an uncomplicated cesarean delivery 2 years prior. Notably, she experienced similar self-resolving symptoms 1 week earlier, during which ultrasonography (USG) demonstrated a viable transverse fetus (heart rate 165–180 bpm) with a 9.4 × 3.0 cm hypoechoic lesion adjacent to the anterior lower uterine segment exhibiting venous flow signals.

Upon this admission, she presented curled up on the bed, appearing miserable and pale with clammy skin and altered consciousness. However, there were no uterine contractions or tenderness on examination. Her blood pressure was measured at 58/34 mm Hg, and her pulse rate was 130 beats per minute. She had no fever, a temperature of 36°C, oxygen saturation of 99% on room air, and a respiratory rate of 24 breaths per minute. There was no vaginal bleeding or fluid loss. Fetal heart was not detected, prompting an immediate bedside scan that revealed a transverse stillbirth without anechoic zone behind the placenta.

Upon admission, necessary blood investigations indicated a hemoglobin level of 7.5 g/dL, creatinine level of 84.8 µmol/L, and normal levels of coagulation factors, serum amylase, serum lipase, and B-type natriuretic peptide. Abdominal computed tomography was performed to locate the bleeding, revealing a bleeding area at the base of the uterus. Within 30 minutes, the hemoglobin level decreased to 6.5 g/dL, raising concerns of severe intrauterine bleeding. Anti-shock therapy and emergency cesarean section were promptly initiated.

**Figure 1.**
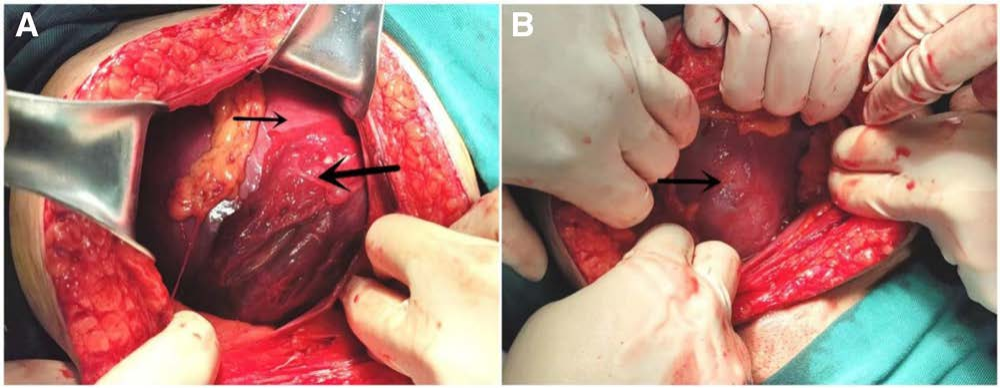
Intraoperative picture showing uterine torsion. (A) Intraoperative image showing the uterus (arrow) and fimbrial portion of right oviduct and parauterine venous plexus (bold arrow). (B) Intraoperative image showing the uterus repositioned to its normal anatomical position.

**Figure 2.**
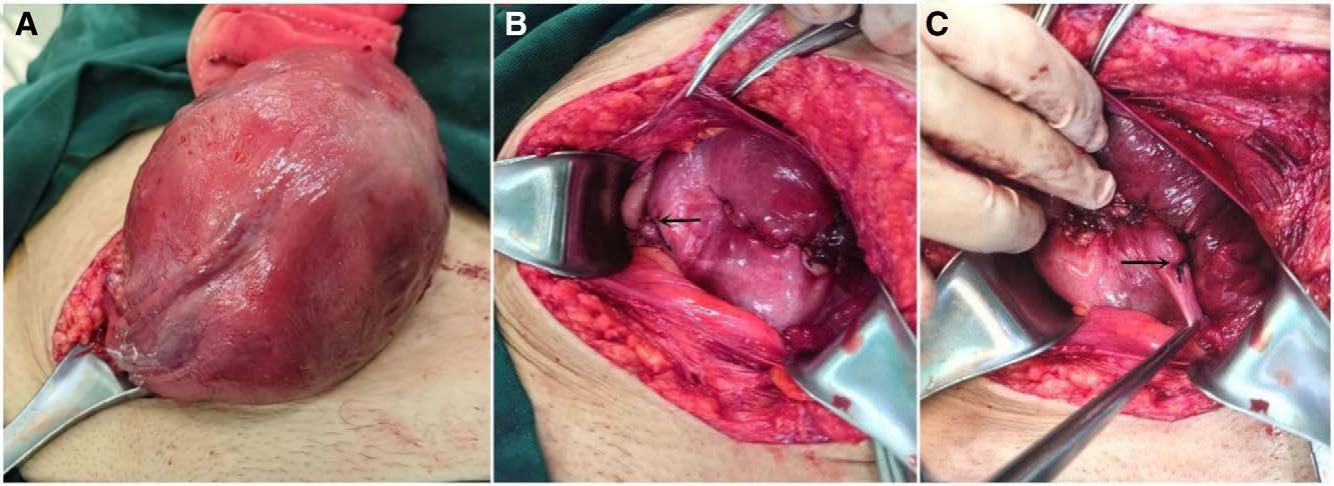
Intraoperative picture showing shortening the round ligament of the uterus. (A) Intraoperative image showing the uterus rotation of about 60° to the left at a relaxed state with long and lax round ligaments. (B) Showing right shortening the round ligament of the uterus. (C) Showing left shortening the round ligament of the uterus.

Under spinal anesthesia, the abdomen was opened through the previous cesarean incision. No blood was observed in the abdomen. The lower uterine segment was found to be covered by the right adnexa, round ligament, and suspensory ligament of the ovary (Fig. 1). A 180° uterine torsion was diagnosed upon careful examination. The surgeon successfully repositioned the uterus to its normal anatomical position by rotating the right uterine horn 180° counterclockwise. A transverse incision was made in the lower uterine segment, and a deceased male infant weighing 2320 g was delivered through breech extraction accompanied by normal amniotic fluid. There were no signs of abruption, and the placenta appeared normal for the gestational age. Following closure of the lower uterine segment incision with a standard two-layer suture, the uterus was temporarily removed from the abdomen to notice the rotation and laxity of ligaments. It rotated approximately 60° to the left at a relaxed state due to long and lax round ligaments (Fig. 2). To prevent postoperative uterine torsion, plication of the round ligament was performed at the conclusion of the procedure. Prior to closing the abdomen, the uterus, parametrium, and bilateral adnexa were observed to be normal.

Due to unstable vital signs, the patient received 4 units of red blood cells prior to the operation. Intraoperatively, no evidence of intrauterine bleeding was noted, and the total blood loss was measured at 400 mL. The postoperative hemoglobin level rose to 10.6 g/dL. The patient recovered well and was discharged on the fourth postoperative day.

## 3. Discussion

Uterine rotation in the third trimester is frequent during a cesarean section. However, the rotation of the uterus by more than 45° along the major axis, defined as uterine torsion, is very rarely observed. Uterine torsion up to 180° and even 270° has been reported in the literature, which is even rarer and can lead to serious adverse pregnancy outcomes.^[1]^ The maternal mortality is 2.0% while the perinatal mortality stands at 38.2%.^[2]^ The first report of uterine torsion in humans was published in 1876.^[3]^ Since then, cases of uterine torsion have been rarely reported. In a report from 1992 including 218 cases,^[4]^ it was indicated that the uterus is dextrorotated in two-thirds of cases, while in this case, the uterus is levorotatory.We conducted a review of the literature and summarized 10 relevant papers (Table 1), which showed that uterine torsion can occur at any gestational age and the clinical manifestations are atypical.^[3,5–13]^

**Table 1:** Results of the literature review.

References	Gestational weeks	History of gynecological surgery or other special conditions	Pregnancy and childbirth	Clinical manifestation	Outcome
Kathleen E Cook et al^[5]^	36+	No	G6P3	Pain and placental abruption amniotic fluid embolus.	Fetal death
P Guié et al^[8]^	37+	No	G3P2	Pain	Fetal death
Munro KI et al^[13]^	32+	No	Not clear	Pain and shock	Fetal death
Wilson D et al^[3]^	36+	No	G3P2	Uterine cramping	Fetal alive
Fatih F F et al^[7]^	22+	No	G1P0	Pain and shock	Fetal death
Ulu I et al^[6]^	37	Cesarean section	G3P2	Pain and mild vaginal bleeding	Fetal alive
Jarosław et al^[11]^	19+	No	G1P0	Pain	Fetal alive
Feng-Ling Yin et al^[9]^	40+	Hysteromyomectomy	Not clear	Pain	Fetal alive
Gao Q et al^[10]^	33+	Multifetation complete bicorporeal uterus	Not clear	Hypotension and uterine cramping	Fetal alive
Gaikwad V et al^[12]^	32+	No	GIP0	Pain	Fetal death

The exact mechanism and etiology of uterine torsion are unknown. Several predisposing factors have been reported,^[14]^ such as uterine fibroids, dysplasia, adhesions, ovarian tumors, pelvic abnormalities, placenta previa, abdominal trauma, abnormal fetal position, abdominal wall relaxation, and uterine ligament relaxation. However, these factors are nonspecific and not always useful in predicting this rare complication of pregnancy.

The clinical manifestations of uterine torsion are atypical, including abdominal pain, nausea, vomiting, oliguria, hematuria, and other urinary symptoms. The severity of symptoms depends on the degree and duration of the torsion. Patients with mild torsion diagnosed during cesarean section may generally have no discomfort.^[14]^ However, severe abdominal pain and shock are often present in critical patients,^[5]^ similar to the clinical manifestations of placental abruption and uterine rupture.

The case reported in this paper has a particular medical history that has not been described before. It occurred a week before admission when the patient developed sudden, untriggered severe abdominal pain with shock, which disappeared without any treatment in 2 hours. In retrospect, uterine torsion had occurred a week before admission, which can explain the approximately 9.4 × 3.0 cm echo-free area located slightly to the left of the anterior wall of the inferior uterine segment. The relief of symptoms may be due to a reduction in the angle of torsion as the patient changed positions. At that time, the placental position reported by ultrasound had changed from the posterior to the anterior wall of the uterus, which was unfortunately not detected in time. If this change had not been missed and uterine torsion had been diagnosed without delay, there should have been no intrauterine fetal death outcome.

Most reported cases of uterine torsion are accompanied by placental abruption.^[6]^ Due to variable symptoms and lack of specific diagnostic signs, uterine torsion is extremely difficult to identify before laparotomy. It is often misdiagnosed before surgery as placental abruption,^[7]^ uterine rupture, appendicitis, or pelvic mass torsion. Each of these misdiagnoses leads to an immediate cesarean section to discover the true cause and avoid maternal and perinatal mortality.^[8]^

It has been reported that computed tomography (CT) scans and magnetic resonance imaging (MRI) can find some signs to assist in diagnosis,^[15]^ such as infarction/ischemia of pelvic neoplasm, gas in the cavity of the uterus, or changes in placenta and pelvic neoplasm position. The uterine whirl sign is the most distinctive and frequent finding on CT scans and MRI, which cannot be seen on USG. However, when the patient is in critical condition, there may not be enough time to complete CT scans and MRIs. In this case, the patient had a CT scan that only revealed a bleeding area in the uterus. Upon reviewing the case data, it was noted that the position of her placenta had changed on USG.

During a cesarean section of the lower uterine segment, it is possible to inadvertently cut the posterior lower segment if uterine torsion is not identified in a timely manner. There have been reports indicating that uterine torsion may not be successfully corrected due to the limitations of a transverse incision and abdominal adhesions, resulting in a transverse posterior hysterotomy.^[13,16]^ However, it is crucial to remain vigilant for the potential occurrence of embolism following successful reduction of uterine torsion. In the case presented, there was no evidence of uterine placental apoplexy, uterine ischemic necrosis, and the patient’s blood pressure was restored after the uterus was repositioned. Furthermore, thorough postoperative examinations did not reveal any signs of embolism in the patient.

The controversy surrounding whether to shorten the round ligament of the uterus persists. Theoretically, shortening the round ligament of the uterus may better maintain the position of the uterus; however, researchers have observed that only 1 out of 33 patients who did not undergo shortening experienced uterine torsion.^[4]^ The patient discussed in our report underwent shortening of the round ligament of the uterus, and there have been no occurrences of uterine torsion during the follow-up period to date.

## 4. Conclusion

The rarity of uterine torsion during pregnancy and the limited clinical experience in its management underscore the clinical significance of documenting each case for improved understanding and evidence-based practice. Uterine torsion must be included in the differential diagnosis when acute abdominal pain and hemodynamic instability occur during pregnancy, particularly in cases where conventional etiologies (e.g., uterine rupture, placental abruption) fail to account for the clinical presentation. Presumptive diagnoses of uterine torsion necessitate expeditious surgical exploration, as time-critical intervention is paramount to mitigate catastrophic maternal–fetal complications including hemorrhagic shock and irreversible ischemic injuries.

## Author contributions

**Conceptualization:** Min Zhou.

**Data curation:** Min Zhou, Yanting Ding.

**Formal analysis:** Linghui Liu, Yanting Ding.

**Investigation:** Linghui Liu.

**Methodology:** Linghui Liu.

**Supervision:** Linghui Liu.

**Visualization:** Min Zhou.

**Writing – original draft:** Linghui Liu, Yanting Ding.

**Writing – review & editing:** Min Zhou, Yanting Ding.
